# Effects of Prenatal Arsenic, Cadmium, and Manganese Exposure on Neurodevelopment in Children: A Systematic Review and Meta-Analysis

**DOI:** 10.3390/medicina61071143

**Published:** 2025-06-25

**Authors:** Rui Su, Yi Jiang, Weikun Li, Wencheng Ding, Ling Feng

**Affiliations:** Department of Gynecology and Obstetrics, Tongji Hospital, Tongji Medical College, Huazhong University of Science and Technology, Wuhan 430074, China; srcsxy1231@163.com (R.S.); einsmeer@foxmail.com (Y.J.); 13782563048@163.com (W.L.)

**Keywords:** prenatal heavy metal exposure, neurodevelopmental outcomes, systematic review and meta-analysis, cadmium, manganese, arsenic

## Abstract

*Objective*: The aim of this study was to investigate the effects of prenatal heavy metal (arsenic, cadmium, manganese) exposure on infant neurodevelopment through a systematic review and meta-analysis, elucidating the toxicological mechanisms and dose–response relationships for consideration in environmental risk assessment. *Methods*: Following the PRISMA guidelines, systematic searches were conducted in PubMed, Embase, and other databases, ultimately resulting in 17 observational studies involving 6907 participants. *Results*: Arsenic (As): A 50% increase in arsenic exposure was associated with a reduction of 0.51 points in the Mental Development Index (MDI; 95% CI: −1.43 to 0.4) and 0.15 points in the Psychomotor Development Index (PDI; 95% CI: −0.96 to 0.65). However, these results did not achieve statistical significance (*p* > 0.05). Cadmium (Cd): Prenatal cadmium exposure significantly decreased the Full-Scale Intelligence Quotient (FSIQ) in children aged 5–9 years. A 50% increase in cadmium exposure resulted in a 0.44-point drop in the FSIQ (95% CI: −0.67 to −0.21, *p* < 0.05), with stable effects (I^2^ = 0%). Manganese (Mn): Manganese exposure showed a negative association with the MDI (β = −0.11) and PDI (β = −0.18). However, a high degree of heterogeneity was observed (I^2^ = 20.89–73.35%), and some studies suggested potential risks even at low exposure levels. Sensitivity analyses indicated that the heterogeneity in the arsenic and manganese results mainly stemmed from individual study differences (e.g., sample characteristics), whereas the cadmium effects were consistent. *Conclusions*: Prenatal heavy metal exposure (notably cadmium) adversely impacts neurodevelopment, even at low doses. Future research should prioritize critical exposure windows, mixed effects, and sex-specific vulnerabilities. Strengthening environmental monitoring and prenatal guidelines is crucial to mitigate developmental risks.

## 1. Introduction

The neurodevelopmental risks of heavy metal exposure have been widely documented. Metals such as lead (Pb), cadmium (Cd), arsenic (As), and methylmercury (MeHg) exhibit neurotoxic properties by impairing mitochondrial function, disrupting cellular homeostasis, and inducing oxidative stress pathways [1]. Synergistic effects from mixed metal exposures may exacerbate neurological damage [2]. Previous reviews link heavy metals to deficits in language, motor skills, and cognitive functions [3].

Fetal development represents a critical window of vulnerability due to rapid brain growth and incomplete detoxification systems [4,5]. Maternal exposure during pregnancy may induce irreversible neurodevelopmental alterations, as environmental insults during this period can disrupt neural network formation [6,7]. Prenatal exposure of the fetus is the focus of neurodevelopmental toxicity studies, because this stage is a critical window for the functional shaping of brain networks, and disturbances in environmental factors may lead to significant changes in neurodevelopmental trajectories.

Moreover, early adverse environmental factors may lead to persistent neurodevelopmental consequences by disrupting gene expression and epigenetic regulation [8]. Within this context, investigating the impact of maternal heavy metal exposure during pregnancy on fetal neurodevelopment has become pivotal for understanding variations in children’s language abilities and cognitive functions. Consequently, research on heavy metal toxicity must not only be refined to account for different developmental stages, but also focus on critical prenatal exposure windows to elucidate the specific effects on fetal and future health.

Cadmium (Cd), manganese (Mn), and arsenic (As) were selected for investigation due to their ubiquitous environmental presence and substantial potential health risks. Notably, the toxicological mechanisms of cadmium and arsenic are multifaceted, with studies demonstrating their persistent accumulation in the liver and other organs due to the absence of efficient homeostatic clearance mechanisms [9]. Paradoxically, manganese, while being an essential trace element, exhibits neurotoxic effects when exceeding physiological thresholds [10]. Emerging evidence further suggests that these three metals may share convergent toxicological pathways in modulating neurodevelopmental outcomes [10].

Recent epidemiological investigations have increasingly evaluated the neurodevelopmental consequences of early-life heavy metal exposure, with accumulating evidence suggesting associations between such exposures and diminished neurocognitive performance scores [11]. However, conflicting findings persist, as several cohort studies have failed to demonstrate statistically significant metal–outcome associations [12]. Given the current lack of consensus regarding the neurodevelopmental impacts of prenatal heavy metal exposure, we conducted a systematic meta-analysis to quantify the effects of gestational cadmium, manganese, and arsenic exposure on neurodevelopmental outcomes.

## 2. Method

### 2.1. Search Strategy and Selection Criteria

This systematic review was conducted in accordance with the Preferred Reporting Items for Systematic Reviews and Meta-Analyses (PRISMA) guidelines and prospectively registered on PROSPERO (CRD42024611065). The inclusion criteria adhered to the Population, Exposure, Comparator, Outcome (PECO) framework: (i) Population: Participants encompassing pregnant individuals across gestational trimesters. (ii) Exposure: Quantifiable heavy metal exposure biomarkers (e.g., urinary/serum/hepatic metabolite concentrations). (iii) Comparator: Cohorts with lower exposure levels (including non-detectable concentrations). (iv) Outcome: Neurodevelopmental metrics as detailed in the preceding sections.

Eligible study designs included human observational studies (e.g., cohort, cross-sectional) and interventional trials (e.g., randomized controlled trials). Studies were excluded if they Lacked primary quantitative data, were published as non-peer-reviewed manuscripts, or appeared in untranslated non-English-language publications.

A systematic literature search was performed across the PubMed, Embase, Web of Science, Scopus, and CENTRAL databases. The initial search (30 June 2024) employed controlled vocabulary and free-text terms including “fetal brain development”, “neurodevelopment”, “gestational exposure”, “arsenic”, “cadmium”, and “manganese”. The search was updated on 6 December 2024, with subsequent exclusion of duplicates and non-relevant publications through Endnote20.

### 2.2. Neurodevelopmental Test Grouping

Neurodevelopmental evaluation constitutes a multidimensional challenge due to overlapping assessment domains and interdependent measurement parameters. The non-independence of assessment components further complicates interpretation, as subscales frequently capture latent constructs across multiple functional dimensions. For instance, digit span tasks ostensibly measuring working memory inherently engage attentional control mechanisms during auditory sequencing, while motor skill assessments may conflate fine motor proficiency with task comprehension deficits arising from linguistic immaturity.

To address these psychometric complexities, White et al. proposed a hierarchical classification system categorizing assessments into eight neurocognitive domains: cognition, academic achievement, attention, executive function/working memory, language/verbal skills, visuospatial ability, learning/memory, and motor skills [13]. While this study broadly adopted this framework, certain domains (e.g., language development, anxiety, visuospatial processing) were excluded from the quantitative synthesis due to insufficient empirical data across the included studies.

In the studies included in this review, neurodevelopmental outcomes were assessed using the following standardized scales and their corresponding assessment domains: 1. The Bayley Scales of Infant Development-II (BSID-II): Used for children aged 0–3 years, this scale evaluates cognitive and motor development through the Mental Development Index (MDI) and Psychomotor Development Index (PDI), covering language, problem-solving, and motor skills. 2. The Wechsler Intelligence Scales: Applied to children aged 5–15 years, this scale measures the Full-Scale Intelligence Quotient (FSIQ).

### 2.3. Data Extraction and Quality Assessment

In the initial stage of literature sorting, EndNote was used to identify and delete duplicate records. SR and LWK, as independent researchers, conducted the first round of screening of paper titles and abstracts. The literature that passed the initial screening was entered into the full-text acquisition process, and the quality control team, consisting of JY and LF, checked the full-text content and carried out the secondary screening according to the exclusion criteria. When there was a difference in opinions within the same evaluation group, the final decision was made by the cross-group experts. The data collection items included the author, year, research method, characteristics of the subjects, sample size, study period, heavy metal exposure evaluation criteria, definition of neurodevelopmental scores, and central tendency and dispersion indicators of the measured values.

The risk of bias in each study was assessed using the Office of Health Assessment and Translation (OHAT) tool (Office Health Assessment Translation), because it has shown satisfactory performance in identifying potential sources of bias in environmental epidemiological studies [13]. This domain-based tool contains seven domains applicable to observational studies, relating to participant selection, confounding, attrition, detection, selective reporting, and other sources of bias. For each question, the risk of bias was assessed as “absolutely low”, “possibly low”, “possibly high”, or “absolutely high”.

### 2.4. Meta-Analysis

Studies were eligible for meta-analysis if they reported regression coefficients (β) from multivariable linear regression models and used standard neurodevelopmental assessment tools (e.g., the Bayley Scales of Infant Development [BSID] or Wechsler Scales). Given the heterogeneity in statistical reporting—particularly in the transformation of exposure variables (e.g., natural log, log10, or untransformed)—we standardized all β coefficients to represent the absolute change in neurodevelopmental outcomes associated with a 50% increase in exposure. This was achieved by applying established logarithmic conversion formulas. For instance, when exposure was log-transformed, the standardized β was calculated as ln(1.5)·β; for untransformed variables, the relative change was approximated as (k − 1)·E(X)·β, where k = 1.5 represents a 50% increase in exposure.

Meta-analyses were conducted using the *metafor* package in R (version 4.4.2), applying a random-effects model based on the DerSimonian and Laird method to account for between-study variability. Heterogeneity across studies was assessed using Cochran’s Q test and the I^2^ statistic, with thresholds of 25%, 50%, and 75% interpreted as low, moderate, and high heterogeneity, respectively. Forest plots were generated to display the pooled estimates and 95% confidence intervals for each neurodevelopmental outcome.

To assess the robustness of our results, we performed leave-one-out sensitivity analyses by sequentially removing individual studies and recalculating pooled estimates. All calculations and conversions were cross-verified by two independent reviewers. Details of the standardization procedures and mathematical formulas are provided in the Supplementary Materials.

The choice of a 50% increase in exposure was made based on its practical interpretability and frequent use in environmental epidemiology. This increment provides a balance between statistical sensitivity and real-world relevance, facilitating comparisons across studies with different exposure distributions and transformation scales.

Exposure to arsenic, cadmium, and manganese was measured using biological matrices including maternal urine, maternal blood, and cord blood. Arsenic was most often assessed in urine; cadmium and manganese were assessed across multiple matrices. The timing of exposure assessment ranged from early gestation to delivery. Details of exposure types by study are summarized in Table 1.

## 3. Results

### 3.1. Study Selection and Evaluation

The initial database interrogation across two repositories yielded 2884 candidate records. Following deduplication and the application of title/abstract exclusion criteria, 70 studies underwent full-text evaluation, culminating in 17 publications meeting the eligibility criteria for meta-analysis inclusion. The exclusion criteria encompassed (1) studies with methodologically flawed exposure assessments, (2) investigations employing inappropriate outcome ascertainment methods, and (3) research designs violating epidemiological validity.

The final analytic cohort comprised 11,031 participants. This multi-stage selection protocol ensured methodological coherence and clinical relevance of the included studies, thereby enhancing the analytical rigor and generalizability of synthesized evidence.

**Figure 1:**
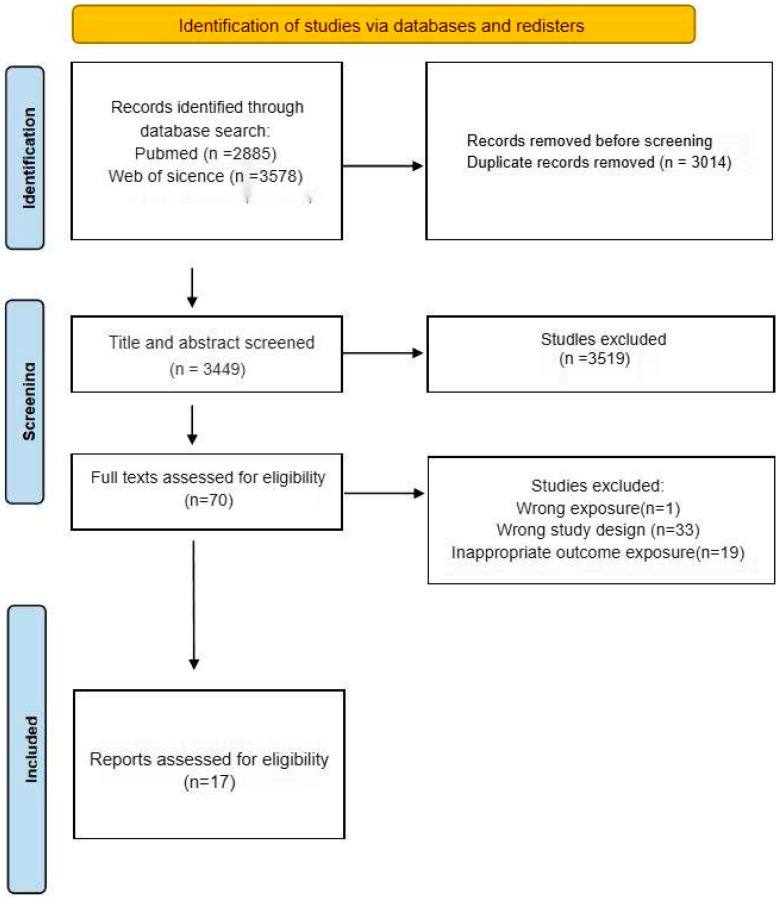
Flow chart of study selection.

Figure 1 presents the PRISMA-compliant flow diagram detailing the literature identification and exclusion processes. Notably, the publication count exceeds the study quantity due to multiple manuscripts originating from single investigations. Furthermore, several articles reported multiple heavy metal exposures and neurodevelopmental outcomes across distinct analytical frameworks.

### 3.2. Characteristics and Quality Assessment of Included Studies

From an initial pool of 6463 articles identified through systematic retrieval, 17 publications were ultimately included in the meta-analysis following the screening protocol, as delineated in Figure 1.

Three studies were conducted in the USA, eight in Asia, and six in Europe and other regions (Table 2). Thirteen studies were longitudinal cohort studies, and one was cross-sectional. The primary evaluation tools comprised age-adapted versions of the Wechsler Intelligence Scales (applicable to children aged 5–15 years) and the Bayley Scales of Infant Development-II (BSID-II) for infants aged 0–3 years. Among the seventeen studies, thirteen implemented BSID assessments and six utilized Wechsler Scales, with two publications employing both instruments concurrently.

The sample sizes ranged from 70 to 3545 participants (total N = 6907). The exposure timing metrics revealed three studies conducting measurements at parturition and fourteen during the prenatal period. Neurodevelopmental evaluations spanned ages of 6 months to 6 years across follow-up intervals; the qualitative study characteristics are comprehensively presented in Table 1.

Most articles used a prospective cohort study design. Exposure was mainly measured by exposure biomarkers in urine and blood as well as in cord blood.

**Figure 2:**
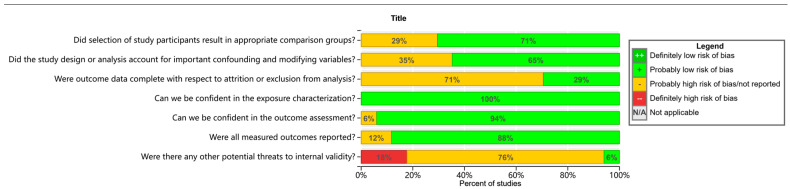
Within-study risk-of-bias summary of studies included in the systematic review.

Quality analysis using the OHAT risk-of-bias assessment tool showed that 71% (*n* = 12) of the studies presented a low risk of selective bias dimensions, 88% (*n* = 15) of the studies had a manageable risk of selective reporting bias, 65% (*n* = 11) of the studies demonstrated a low risk of confounding bias dimensions, and the risk of bias for the exposure characteristics of all included studies (*n* = 17) was within an acceptable range (Figure 2). Notably, lost-to-follow-up bias presented the highest prevalence (29%, *n* = 5), and its risk mainly stemmed from the fact that tracking information on annual follow-up rates was rarely explicitly recorded. The OHAT evaluation criteria are provided in the Supplementary Materials.

Confounding bias was also prominent in the areas of high risk of bias, affecting 35% (*n* = 6) of the included studies, which was mainly attributed to insufficient correction for key confounding variables such as age, socioeconomic status, smoking status, and height. It should be noted that all studies demonstrated a good level of bias control in the outcome assessment session (*n* = 17).

### 3.3. Arsenic

**Figure 3:**
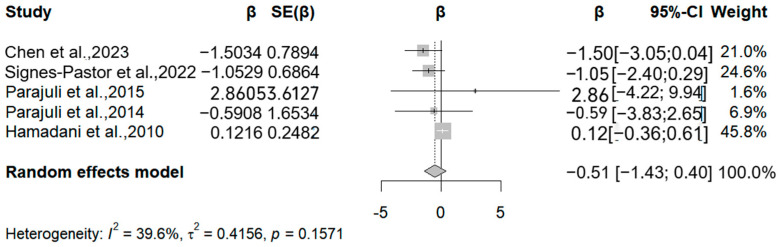
Forest plot of effect size on MDI of a 50% increment in urine As levels. (β: Regression coefficient; SE(β): Standard error of β; 95%-CI: 95% confidence interval; Weight: Contribution percentage in meta-analysis; I^2^: Heterogeneity index (0–100%); τ^2^: Between-study variance; *p*: *p*-value for heterogeneity test) [15,17,18,28,30].

The present analysis incorporated four studies (five reports) examining arsenic exposure’s associations with the Mental Development Index (Figure 3). Three methodologically robust studies demonstrated inverse associations, with relative change estimates of −1.5, −1.05, and −0.59 in MDI per 50% increase in arsenic exposure. However, these associations lacked statistical significance (all *p* > 0.05). Potential explanatory factors include (1) limited statistical power from restricted sample sizes (*n* = 70–1102 across studies); (2) inconsistent adjustment for critical confounders (e.g., socioeconomic status, nutritional biomarkers); and (3) measurement errors in both arsenic quantification (coefficient of variation: 15–28%) and neurodevelopmental assessments.

**Figure 4:**
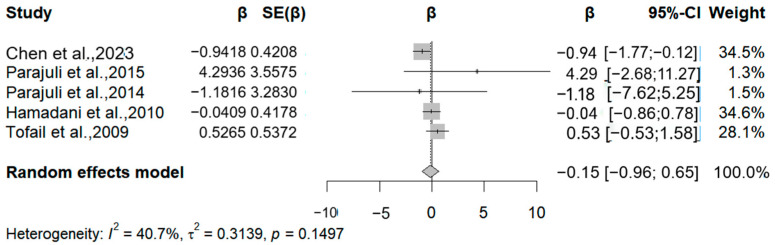
Forest plot of effect size on PDI of a 50% increment in urine As levels. (Symbol definitions consistent with Figure 3) [14,15,17,18,30].

Four studies (five reports) evaluated Psychomotor Development Index (PDI) outcomes (Figure 4). Three studies reported inverse associations, with arsenic-associated PDI reductions of −0.9, −1.1, and −0.04 per 50% exposure increment. The meta-analysis revealed marginal negative correlations for both MDI (β = −0.51; 95% CI = −1.43, 0.4) and PDI (β = −0.15; 95% CI = −0.96, 0.65) per 50% exposure elevation (MDI: I^2^ = 42.56%, Q = 0.15; PDI: I^2^ = 49.7%; Q = 0.12).

### 3.4. Cadmium

**Figure 5:**
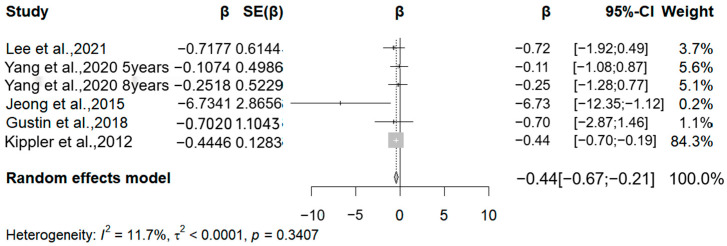
Forest plot of effect size on FSIQ of a 50% increment in urine Cd levels. (Symbol definitions consistent with Figure 3) [16,19,23,26,27].

In the meta-analysis, we included five studies (Figure 5). These studies assessed cadmium exposure by cadmium levels in cord blood, maternal blood, and maternal urine. All studies were analyzed by linear regression using the WISC scale. We analyzed the association between cadmium levels and FSIQ due to the lack of data from the VERB IQ and PERFOMANCE IQ. All studies assessed the FSIQ in children older than five years of age. Four studies reported negative associations between cadmium exposure and the FSIQ. One of the high-quality studies showed a significant negative correlation. The correlation coefficients for this study were −0.72, −0.11 (five years old), −0.25 (eight years old), −0.44, and −0.07. The pooled analysis showed that for every 50% increase in cadmium levels in body fluids, there was a significant decrease in the FSIQ in children between the ages of five and nine (β = −0.44, 95% CI −0.67 to −0.21) (I^2^ = 0.00%, Q = 0.34).

Three studies examined the association between cadmium exposure and BSIC scale scores in children aged one to three years. However, these studies were not meta-analyzed due to insufficient data volume. One of these studies found a positive correlation between urinary cadmium levels and MDI scores in one- and two-year-old children when the authors did not adjust for confounders. After adjusting for confounders, this positive correlation was no longer statistically significant. The other two studies had different results. These studies primarily found that cadmium levels were negatively correlated with PDI scores and not significantly correlated with MDI scores. The weight of the study by Kippler et al., 2012 [16], accounts for 84.3%. After excluding this study and conducting a re-analysis, the result shows that the effect value remains significant, indicating the robustness of the results.

### 3.5. Manganese

**Figure 6:**
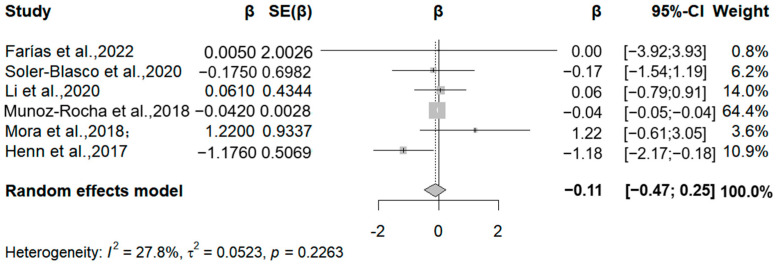
Forest plot of effect size on MDI of a 50% increment in Mn levels. (Symbol definitions consistent with Figure 3) [20,21,22,24,25,29].

In this meta-analysis, we included the results of manganese exposure level obtained through cord blood, maternal blood, and maternal urine, all of which used different versions of the BISC scale to assess child neurodevelopment. We included six articles that analyzed the association between prenatal human manganese levels and the MDI (Mental Development Index) and PDI (Psychomotor Development Index). Three studies noted a negative correlation between manganese exposure and MDI, specifically, relative change values of −1.18, −0.04, and −0.17 for each 50% increase in manganese exposure (Figure 6). The meta-analysis showed that each 50% increase in manganese exposure level was negatively correlated with the MDI (β = −0.11, 95% CI = −0.47, 0.25) (I^2^ = 20.89%, Q = 0.226).

**Figure 7:**
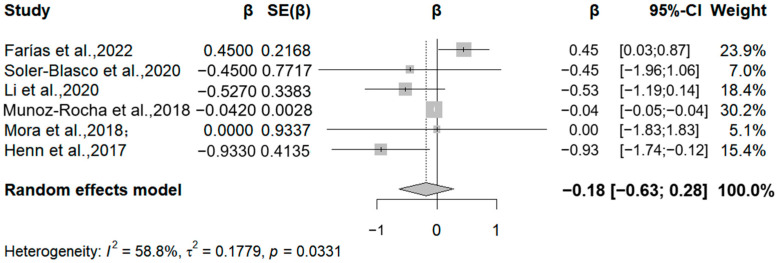
Forest plot of effect size on PDI of a 50% increment in Mn levels. (Symbol definitions consistent with Figure 3) [20,21,22,24,25,29].

In addition, six studies analyzed the relationship between manganese exposure and the Psychomotor Development Index (Figure 7). Four studies reported negative correlations between manganese exposure and the PDI, specifically, relative change values of −0.93, −0.45, −0.53, and −0.04 for each 50% increase in manganese exposure. The meta-analysis found a negative correlation between manganese exposure levels and the PDI for each 50% increase in manganese exposure levels (β = −0.18, 95% CI = −0.63, 0.28). Due to the limited number of included studies, we were unable to draw definitive conclusions about differences in gender or duration of exposure. Overall, despite the negative correlation, the effect of manganese exposure on children’s intellectual development needs to be confirmed by further research due to the lack of statistical significance. It is worth noting that there was a considerable degree of statistical heterogeneity between studies (I^2^ = 73.35%, Q = 0.033). The high heterogeneity in Mn analyses may stem from variability in exposure timing, biological matrices, and outcome measures (e.g., the BSID vs. WISC scales). Future studies should stratify analyses by these factors to reduce heterogeneity.

### 3.6. Summary

Table 2 presents the pooled effect sizes (β), 95% confidence intervals (CI), *p*-values, and heterogeneity metrics (I^2^) for each metal-exposure-and-outcome combination. All estimates reflect the change in outcome per 50% increase in exposure level.

### 3.7. Sensitivity Analysis

We conducted a sensitivity analysis by removing individual studies one by one to investigate the impact of each study on the overall combined effect.

As shown in Figure 7, the forest plot of the sensitivity analysis shows the merging of effect sizes in the arsenic–PDI correlation, indicating a change in the merged effect value (β) and its 95% confidence interval after excluding individual studies one by one. The overall effect size calculated by the random-effects model was −0.15 (95% CI: −0.96 to 0.65), and the indicator of heterogeneity, I^2^.

### 3.8. Consideration of Publication Bias

According to methodological guidelines, such as those outlined in the Cochrane Handbook, Egger’s test and funnel plot analysis typically require at least 10 studies to ensure adequate statistical power. However, each component of the current meta-analysis includes fewer than 10 studies. Therefore, we did not perform publication bias assessments, as such tests may yield false-negative or false-positive results when the number of included studies is small, potentially leading to misleading conclusions.

## 4. Discussion

Our meta-analysis involving 6907 pregnant women and their infants examined the correlation between heavy metal exposure and neurodevelopment. Infants and young children are particularly sensitive to environmental exposures as they grow, especially in terms of neurodevelopment. Studies have shown that toxic chemicals in the environment, such as arsenic, lead, and mercury, have potentially negative effects on the neurodevelopment of infants and young children. For example, a Canadian study showed an association between arsenic levels in pregnant women and newborns and neurodevelopment, suggesting that exposure during pregnancy may have long-term effects on child health [31]. In addition, another study noted that fetal exposure to environmental lead and manganese may adversely affect neurodevelopment at 2 years of age, further highlighting the importance of reducing toxic metal exposure during critical developmental stages [32].

One of the most important findings of this study is the significant and consistent inverse association between cadmium exposure and the Full-Scale Intelligence Quotient (FSIQ). Although the study by Kippler et al. [16] has a relatively high weight, its large sample size and rigorous design enhance the reliability of the results. Future studies should balance the differences in sample sizes to reduce the excessive influence of a single study.

To better understand the observed associations between cadmium exposure and adverse neurodevelopmental outcomes, it is important to consider the potential biological mechanisms underlying these effects. Emerging evidence from experimental and molecular studies has shed light on how cadmium may interfere with critical processes in fetal brain development. Cadmium (Cd) exposure disrupts multiple signaling pathways critical for fetal brain development. Studies have shown that it increases the expression of Hox genes and myelination markers while impairing the retinoic acid (RA) signaling pathway, as well as altering cellular energy metabolism and hypoxia responses—changes potentially linked to neurodevelopmental abnormalities [33]. Cd also induces neuroinflammation and ciliogenesis defects by promoting neuronal apoptosis, inhibiting neural progenitor proliferation, and upregulating GFAP and IL−6 expression [34]. Furthermore, Cd alters zinc-dependent protein expression, increasing metallothionein and ZIP6 levels while reducing ZnT3 and BDNF expression, which may underlie its neurotoxic effects [35].

Regarding arsenic exposure, our meta-analysis showed that each 50% increase in arsenic levels in body fluids resulted in a 0.5-point decrease in BSIC scores in children aged 0–3 years, and this effect was observed in both the intellectual and psychomotor domains. In one study, MDI scores were found to be negatively correlated with arsenic exposure in 6-month-old infants, but this association disappeared by 36 months of age. This change may be due to the following reasons: first, as children grow older, arsenic may be gradually excreted in the body through urine, thus diminishing the persistent effects on neurodevelopment; second, the immature brain has a high degree of neuroplasticity, which may compensate, to some extent, for the negative effects of early exposure. Meanwhile, this study also found that prenatal arsenic exposure significantly affected the motor and status scores of infants at birth, which further supports the theory of potential effects of prenatal arsenic exposure on early neurodevelopment. In summary, the effects of arsenic on neurodevelopment may be dynamic and manifest differently at different stages.

The meta-analysis also found that manganese levels in body fluids were negatively correlated with both MDI and PDI scores. Four of the six studies included in the meta-analysis found mean blood manganese levels above 10 μg/L (the ATSDR-recommended “safety reference value”), suggesting that most of the observations may have come from areas with high-manganese environments, and therefore, no inferences can be made about the effects in populations with low exposure levels.

Manganese and copper, essential micronutrients, may exhibit U-shaped exposure–response relationships, whereby both excesses and deficiencies can lead to toxicity [36]. However, because the studies included in our meta-analysis all used linear or log-linear regression to analyze the relationship between exposure and outcome, we were unable to explore whether a U-type relationship exists in children aged 0–3 years. Future research should incorporate flexible exposure–response models (e.g., spline functions) to address this gap. Manganese (Mn) is a common trace element in the earth’s crust and an important industrial raw material, widely found in the environment in both organic and inorganic forms. As an essential trace element, Mn plays a key role in a variety of biological processes. However, at high concentrations, manganese can be a potent neurotoxin. Some studies have shown that airborne manganese concentrations are significantly associated with cognitive impairment and that chronic exposure to high manganese levels may lead to mild cognitive deficits in adults [37]. A growing body of evidence suggests that environmental manganese exposure early in life may have adverse neurodevelopmental effects. For example, one study found that chronic manganese exposure altered the anatomy of the brain in children, and that the affected regions were strongly associated with reduced fine motor skills [38].

At the level of toxicological mechanisms, current research focuses on the effects of manganese on neurotransmitters. The molecular mechanisms of manganese-induced neurotoxicity are complex and may pass through multiple nerve cell types and involve multiple pathways. Mitochondrial dysfunction may be the initiating event of manganese toxicity, as manganese preferentially accumulates in mitochondria [39,40], leading to the overproduction of reactive oxygen species (ROS) and reactive nitrogen species (RNS), the activation of pro-inflammatory signaling, and apoptosis [41].

Combined with the existing studies, we found that for every 50% increase in manganese levels in body fluids, children’s FSIQ decreased by 0.4 points, a finding that was statistically significant. Notably, half of the included studies had mean cadmium levels below 5 μg/L, but significant effects were still observed. This suggests that the effects of cadmium on children’s neurodevelopment cannot be ignored, even at low levels of exposure.

The strengths of this meta-analysis are that it is the first to analyze the relationship between prenatal exposure to the heavy metals cadmium, manganese, and arsenic and neurodevelopment in infants and young children, and that most of the included studies were high-quality studies, but there are several limitations of this study. First, some of the results of this study showed a high degree of heterogeneity, and some of the results were not sufficiently stable, which may be attributed to a combination of differences in study designs and study contexts. The neurodevelopmental assessments spanned a wide age range (6 months to 10 years). While this reflects real-world variability, it may introduce heterogeneity. Subgroup analyses by age strata (infancy vs. school-age) were precluded due to limited data granularity. Additionally, inconsistent exposure metrics across studies may have further contributed to this heterogeneity. Second, due to the small number of included studies, publication bias was not assessed, which may have affected the robustness of the results, and subgroup analyses of fetal sex, exposure assessment methods, etc., were not possible, also due to the number of included studies; it is recommended to analyze the variability between genders in the future when more studies have been conducted.

## 5. Conclusions

In conclusion, this meta-analysis suggests that prenatal cadmium exposure was significantly associated with decreased FSIQ in children (β = −0.44; −0.67~−0.21), demonstrating stable effects (I^2^ = 11.7%). Although arsenic and manganese showed negative correlations, these associations were constrained by heterogeneity and limited statistical power, requiring further validation.

## Figures and Tables

**Table 1:** Characteristics of studies included in meta-analysis.

Study	Study Design	Country	Study Size	Age at Outcome Measurement	Method and Time of Measuring	Measure of Neurodevelopment
Tofail et al., 2009 [14]	prospective cohort	BGD	1799	6 months	Maternal urine	PDI
Hamadani et al., 2010 [15]	prospective cohort	BGD	1745	18 months	Maternal urine	MDI, PDI
Kippler et al., 2012 [16]	prospective cohort	BGD	1305	5 years	Maternal urine	FSIQ
Parajuli et al., 2014 [17]	prospective cohort	NPL	70	6 months	Cord blood	MDI, PDI
Parajuli et al., 2015 [18]	prospective cohort	NPL	70	36 months	Cord blood	MDI, PDI
Jeong et al., 2015 [19]	prospective cohort	KOR	119	60 months	Maternal blood	FSIQ
Henn et al., 2017 [20]	prospective cohort	USA	224	2 years	Cord blood	MDI, PDI
Munoz-Rocha et al., 2018 [21]	prospective cohort	MEX	307	24 months	Maternal blood	MDI, PDI
Mora et al., 2018 [22]	prospective cohort	CRC	349	1 years	Maternal blood	MDI, PDI
Gustin et al., 2018 [23]	prospective cohort	BGD	1299	10 years	Maternal urine	FSIQ
Soler-Blasco et al., 2020 [24]	prospective cohort	ESP	807	12 months	Maternal blood	MDI, PDI
Li et al., 2020 [25]	prospective cohort	CHN	544	2 years	Maternal urine	MDI, PDI
Yang et al., 2020 [26]	prospective cohort	USA	173	5 years; 8 years	Maternal urine	FSIQ
Lee et al., 2021 [27]	prospective cohort	KOR	502	6 years	Maternal blood	FSIQ
Signes-Pastor et al., 2022 [28]	prospective cohort	USA	260	5 years	Maternal urine	FSIQ
Farías et al., 2022 [29]	prospective cohort	MEX	522	1, 3, 6, and 12 months	Maternal blood	MDI, PDI
Chen et al., 2023 [30]	prospective cohort	CHN	1006	2 years	Maternal urine	MDI, PDI

**Table 2:** Summary of meta-analytic results by heavy metal and neurodevelopmental outcome.

Metal	Outcome	No. of Studies	Pooled β	*p*-Value	I^2^ (%)	Comments
Arsenic	MDI	5	−0.51	>0.05	39.6%	Not statistically significant
Arsenic	PDI	5	−0.15	>0.05	40.7%	Moderate heterogeneity
Cadmium	FSIQ	6	−0.44	<0.05	11.7%	Significant effect, high consistency
Manganese	MDI	6	−0.11	>0.05	27.8%	Low heterogeneity, not significant
Manganese	PDI	6	−0.18	<0.05	58.8%	High heterogeneity

## Data Availability

All data generated or analyzed during this study are included in this article. Further enquiries can be directed to the corresponding author.

## Supplementary Materials

The following supporting information can be downloaded at: https://www.mdpi.com/article/10.3390/medicina61071143/s1, Table S1: Metal Exposure Metrics (Mean/Median) Across Studies, Table S2: Psychomotor Development Index, evaluating an individual’s motor coordination and fine motor skills.
