# Supplementary material for: Effects of Prenatal Arsenic, Cadmium, and Manganese Exposure on Neurodevelopment in Children: A Systematic Review and Meta-Analysis
Source: Medicina (Kaunas). 2025 Jun 25;61(7):1143. doi: 10.3390/medicina61071143 (PMC12300850; doi:10.3390/medicina61071143)
Supplement: Supplementary file 1 [file medicina-61-01143-s001.zip › medicina-3573003-supplementary.pdf]

1.The specific process and principle of effect size transformation

Specifically, in linear regression models where exposure variables were natural logarithm-transformed, we define $c \cdot \beta$ as the absolute change in the response variable corresponding to a c log-unit difference in the exposure variable (i.e., when $\ln(X_1) - \ln(X_0) = c$). Furthermore, the relative change in the original variable X is expressed as $X_1/X_0 = k$, where k represents the multiplicative increase in X. By applying logarithmic properties to this expression, we derive:$\ln(X_1)-\ln(X_0) = \ln(k)$.Thus, $\ln(k) \cdot \beta$ represents the absolute change in the outcome variable. Similarly, for base-10 logarithm-transformed variables, $\log_{10}(k) \cdot \beta$also corresponds to the absolute change. For untransformed studies, we determined the absolute change associated with a mean relative change in the exposure variable equal to k, calculated as $(k - 1) \cdot E(X) \cdot \beta$.

Consequently, we standardized the expression of outcome variables across all studies to reflect the same relative change. In this analysis, k was defined as 1.5, equivalent to a 50% increase in exposure, to The specific process and principle of effect size transformationquantify the absolute change in the outcome variable per 50% exposure increment.

2.OHAT evaluation criteria

Question Format:

• Background

o Definition of the general category of bias

o Clarifying text to explain what study aspects are relevant

o Available empirical information about the direction and magnitude of the bias

o Information about other internal validity assessment tools that consider this element

• Specific risk-of-bias rating instructions customized to each study type

o Detailed criteria are outlined that define aspects of the study design, conduct, and reporting required to reach each risk-of-bias rating

o The criteria are focused on distinguishing among the 4 risk-of-bias answers or ratings (e.g.,outlining factors that separate “definitely low” from “probably low” risk of bias

Answer Format:

Definitely Low risk of bias:

There is direct evidence of low risk-of-bias practices (May include specific examples of relevant low risk-of-bias practices)

Probably Low risk of bias:

There is indirect evidence of low risk-of-bias practices OR it is deemed that deviations from low risk-of-bias practices for these criteria during the study would not appreciably bias results, including consideration of direction and magnitude of bias.

Probably High risk of bias:

There is indirect evidence of high risk-of-bias practices OR there is insufficient information (e.g., not reported or “NR”) provided about relevant risk-of-bias practices

Definitely High risk of bias:

There is direct evidence of high risk-of-bias practices (May include specific examples of relevant high risk-of-bias practices)

The system for answering each risk-of-bias question requires reviewers to choose between low and highrisk-of-bias options. This 4-point scale is based on the approach taken by the Clarity Group at

McMaster University without an answer for mixed or unclear evidence (2013). A conservative approach is taken wherein insufficient information to clearly judge the risk of bias for an individual question results in an answer rating of “Probably High” risk of bias. To clearly identify answers that were reached due to insufficient information, there are two separate symbols for “Probably High” risk of bias: 1) “-“ for indirect evidence of high risk-of-bias practices, and 2) “NR” or not reported when there is insufficient information. The general answer format was adapted from (Koustas et al. 2013).

3.TableS1 Metal Exposure Metrics (Mean/Median) Across Studies.

Study	Country	Route and time of exposure	Metal concentration(μg/L)
Tofail et al.,2009(As)	BGD	Maternal urine(third trimester of pregnancy)	Median:81(37-207)<br>Mean:NR
Hamadani et al.,2010(Mn)	BGD	Maternal urine (GW8 and 30)	Median: GW8 (81) and GW30(84)<br>Mean:NR
kippler et al.,2012(Cd)	BGD	Maternal urine(GW8, on average)	Median:NR<br>Mean:0.27
Parajuli et al.,2014(As)	NPL	cord blood	Median:NR<br>Mean:NR
Parajuli et al.,2015(As)	NPL	cord blood	Median:NR<br>Mean:NR
Jeong et al.,2015(Cd)	KOR	Maternal blood (early pregnancy)	Median:1.49<br>Mean:NR
Henn et al.,2017(Mn)	USA	cord blood	Median:2.3 (1.7–3.3)<br>Mean:NR
Munoz-Rocha et al.,2018(Mn)	MEX	Maternal blood (the 3rd trimester)	Median:27.7（SD=8.7）<br>Mean:NR
Mora et al.,2018(Mn)	CRC	Maternal blood(NR)	Median:24.4±6.2<br>Mean:NR
Gustin et al.,2018(Cd)	BGD	Maternal urine (GW8, on average)	Median:NR<br>Mean:0.2667
Soler-Blasco et al.,2020(Mn)	ESP	Maternal blood(first trimester of pregnancy)	Median:1.50(1.48-1.53)<br>Mean:NR
Li et al.,2020(Mn)	CHN	Maternal urine (before delivery)	Median:1.50 ± 3.26<br>Mean:NR
Yang et al.,2020(Cd)	USA	Maternal urine (GW26)	Median:NR<br>Mean:0.17
Lee et al.,2021(Cd)	KOR	Maternal blood (the second trimester)	Median:NR<br>Mean:0.64
Signes-Pastor et al.,2022(As)	USA	Maternal urine (GW16 and 26)	Median:3.63 (3.19–4.06)<br>Mean:NR
Farías et al.,2022(Mn)	MEX	Maternal blood(the third trimester)	Median:10<br>Mean:NR
chen et al.,2023(As)	CHN	Maternal urine (within GW16)	Median:15.59<br>Mean:16.37

4.Table S2 Psychomotor Development Index, evaluating an individual's motor coordination and fine motor skills.

Scale	Index	Description
WISC	FSIQ	Full Scale IQ, representing an individual’s overall performance across different cognitive tasks.
BISD	MDI	Mental Development Index, assessing an individual's cognitive and psychological development level.
BISD	PDI	Psychomotor Development Index, evaluating an individual's motor coordination and fine motor skills.
